# Proportional modes versus pressure support ventilation: a systematic review and meta-analysis

**DOI:** 10.1186/s13613-018-0470-y

**Published:** 2018-12-10

**Authors:** Jun Kataoka, Akira Kuriyama, Yasuhiro Norisue, Shigeki Fujitani

**Affiliations:** 1 Department of Pulmonary and Critical Care Medicine, Tokyo Bay Urayasu Ichikawa Medical Center, 3-4-32 Todaijima, Urayasu, 2790001 Japan; 2 Emergency and Critical Care Center, Kurashiki Central Hospital, 1-1-1 Miwa, Kurashiki, Okayama 7108602 Japan; 3 Department of Emergency Medicine and Critical Care Medicine, St. Marianna University, 2-16-1 Sugao, Miyamae-ku, Kawasaki, 2168511 Japan

**Keywords:** Pressure support ventilation, Proportional assist ventilation, Neurally adjusted ventilator assist, Asynchrony index, Ventilator weaning, Systematic review, Meta-analysis

## Abstract

**Background:**

Proportional modes (proportional assist ventilation, PAV, and neurally adjusted ventilatory assist, NAVA) could improve patient–ventilator interaction and consequently may be efficient as a weaning mode. The purpose of this systematic review is to examine whether proportional modes improved patient–ventilator interaction and whether they had an impact on the weaning success and length of mechanical ventilation, in comparison with PSV.

**Methods:**

We searched PubMed, EMBASE, and the Cochrane Central Register of Controlled Trials from inception through May 13, 2018. We included both parallel-group and crossover randomized studies that examined the efficacy of proportional modes in comparison with PSV in mechanically ventilated adults. The primary outcomes were (1) asynchrony index (AI), (2) weaning failure, and (3) duration of mechanical ventilation.

**Results:**

We included 15 studies (four evaluated PAV, ten evaluated NAVA, and one evaluated both modes). Although the use of proportional modes was not associated with a reduction in AI (WMD − 1.43; 95% CI − 3.11 to 0.25; *p *= 0.096; PAV—one study, and NAVA—seven studies), the use of proportional modes was associated with a reduction in patients with AI > 10% (RR 0.15; 95% CI 0.04–0.58; *p *= 0.006; PAV—two studies, and NAVA—five studies), compared with PSV. There was a significant heterogeneity among studies for AI, especially with NAVA. Compared with PSV, use of proportional modes was associated with a reduction in weaning failure (RR 0.44; 95% CI 0.26–0.75; *p *= 0.003; PAV—three studies) and duration of mechanical ventilation (WMD − 1.78 days; 95% CI − 3.24 to − 0.32; *p *= 0.017; PAV—three studies, and NAVA—two studies). Reduced duration of mechanical ventilation was found with PAV but not with NAVA.

**Conclusion:**

The use of proportional modes was associated with a reduction in the incidence with AI > 10%, weaning failure and duration of mechanical ventilation, compared with PSV. However, reduced weaning failure and duration of mechanical ventilation were found with only PAV. Due to a significant heterogeneity among studies and an insufficient number of studies, further investigation seems warranted to better understand the impact of proportional modes.

*Clinical trial registration* PROSPERO registration number, CRD42017059791. Registered 20 March 2017

**Electronic supplementary material:**

The online version of this article (10.1186/s13613-018-0470-y) contains supplementary material, which is available to authorized users.

## Introduction

The separation of patient from the mechanical ventilator can take long time, and it is an important phase during the patient ventilator assistance [1]. Pressure support ventilation (PSV) is the most commonly used mode for the liberation process [2, 3], but presents several limitations. First, the optimal pressure support level for weaning varied among patients. Both over- and under-assistance may cause a diaphragm weakness [4]. Critical illness-associated diaphragm weakness is often associated with difficult weaning and prolonged duration of mechanical ventilation [5–7]. Second, PSV can often cause patient–ventilator asynchrony due to the mismatch between pressure support and the patient’s inspiratory demand or effort level [8]. A recent study showed that the presence of asynchrony was associated with prolonged duration of mechanical ventilation and led to increased mortality [9].

Proportional assist ventilation (PAV) [10] and neurally adjusted ventilatory assist (NAVA) [11] are designed to improve patient–ventilator interaction [12]. Both modes are designed to adjust inspiratory pressure proportionally to the patient’s inspiratory demand and are known as proportional modes [13]. PAV+ (Puritan Bennett 840/980 ventilator; Covidien, Boulder, Colorado, USA) automatically measures the elastance and resistance of the respiratory system during spontaneous breathing and delivers the adequate pressure needed to meet the flow and volume demand that are instantaneously measured on a breath-to-breath basis [14]. On the other hand, NAVA (Maquet Critical Care SA, S.lna, Sweden) is controlled by the change of electrical activity of the diaphragm (EAdi) which is obtained by the placement of a nasogastric tube equipped with EMG electrodes [11]. Previous studies have demonstrated that both modes improve the patient–ventilator interaction [15]. Although proportional modes may be efficient as a weaning mode, they have not been examined on a large-scale randomized controlled trial compared to PSV as a weaning mode.

Therefore, we conducted a systematic review and meta-analysis to examine whether proportional modes improve patient–ventilator interaction and whether they have an impact on the weaning success and length of mechanical ventilation in mechanically ventilated patients, in comparison with PSV.

## Methods

Our study protocol was registered at PROSPERO (CRD42017059791) on March 20, 2017. We complied with the PRISMA (Preferred Reporting Items for Systematic Reviews and Meta-analyses) statement for reporting this systematic review [16].

### Eligibility criteria

We included both parallel-group and crossover randomized studies that examined the efficacy of proportional modes (PAV and NAVA) in comparison with PSV in mechanically ventilated adults. We excluded studies that did not examined asynchrony index [17], pediatric and noninvasive ventilation studies, as well as parallel-group studies that applied proportional modes only for spontaneous breathing trial.

### Database search

We searched PubMed, EMBASE, and the Cochrane Central Register of Controlled Trials for eligible articles. Our search strategy was listed in Additional file 1: Table S1. Two authors (JK and AK) independently screened articles retrieved by the search and selected eligible articles. We also inspected the references of included studies for potentially relevant studies. In case of disagreement, the same authors consulted with a third author (YN). We placed no restrictions regarding publication status and languages. Our last search update was May 13, 2018.

### Data extraction and bias assessment

Two authors (JK and AK) independently extracted the following data: (1) participants (age and proportion of females); (2) characteristics (country, type of ICUs, inclusion criteria of participants, parallel or crossover studies); (3) interventions (NAVA or PAV), and (4) outcomes of our interest listed below.

The same authors also independently assessed the risk of bias using Cochrane Risk of Bias tool [18]. Disagreement was resolved through discussion.

### Outcomes

Our primary outcomes were (1) asynchrony index (AI), (2) weaning failure, and (3) duration of mechanical ventilation. The primary outcomes were analyzed for overall proportional modes of ventilation including NAVA and PAV together. AI was described in two different ways in the included studies. AI was either defined as a continuous outcome or as the number of patients with AI > 10%. We thus presented both definitions in our study. Weaning failure was generally defined as the need for switching to a controlled mode or reintubation after extubation. However, there is a significant heterogeneity in the definition of weaning failure among the studies. We thus included only studies with the definition of weaning failure of “extubation failure leading to reintubation.” Our secondary outcomes included (1) weaning time from randomization, (2) switching again to a controlled mode, (3) length of stay in ICU, (4) length of stay in hospital, (5) ICU mortality, (6) in-hospital mortality, (7) patients who needed tracheostomy, (8) incidence of reintubation, and (9) those who required noninvasive ventilation after extubation.

### Statistical analysis

We calculated risk ratios (RRs) for dichotomous outcomes and weighted mean difference (WMD) for continuous outcomes and presented the results with associated 95% confidence intervals (CIs). Since many of the included studies reported continuous outcomes in medians and interquartile ranges, we converted these values to means and standard deviations using a method proposed by Wan et al. [19]. We pooled the data using a random-effects model [20]. There is no established method of pooling crossover studies. However, the pooled outcome of crossover studies is generally conservative than that of parallel studies. Although we acknowledge the unit-of-analysis error (double- or triple-counting studies and participants), we pooled all crossover studies as if they were parallel studies [18]. There is one three-way crossover study [15], and we evaluated the impact of this study by pooling only one comparison at a time in a sensitivity analysis. We assessed statistical heterogeneity with *I*^2^ and *Q* statistics [21]. We did not evaluate small study effect or publication bias according to the Cochrane methodology, because the number of studies included for each analysis was less than ten [18].

We conducted subgroup analysis by the type of intervention, namely NAVA and PAV, and examined the difference of outcomes between these subgroups with test of interaction. We also conducted sensitivity analysis, by excluding unclear or high risk of bias in sequence generation, allocation concealment, blinding of assessors, incomplete outcome reporting, and selective outcome reporting to assess the robustness of our primary outcome analyses.

We performed all analyses with Stata SE, version 15.0 (Stata, College Station, TX, USA). A threshold for statistical significance was set at *p *< 0.05 (two-tailed).

## Results

### Overview of included studies

**Fig. 1:**
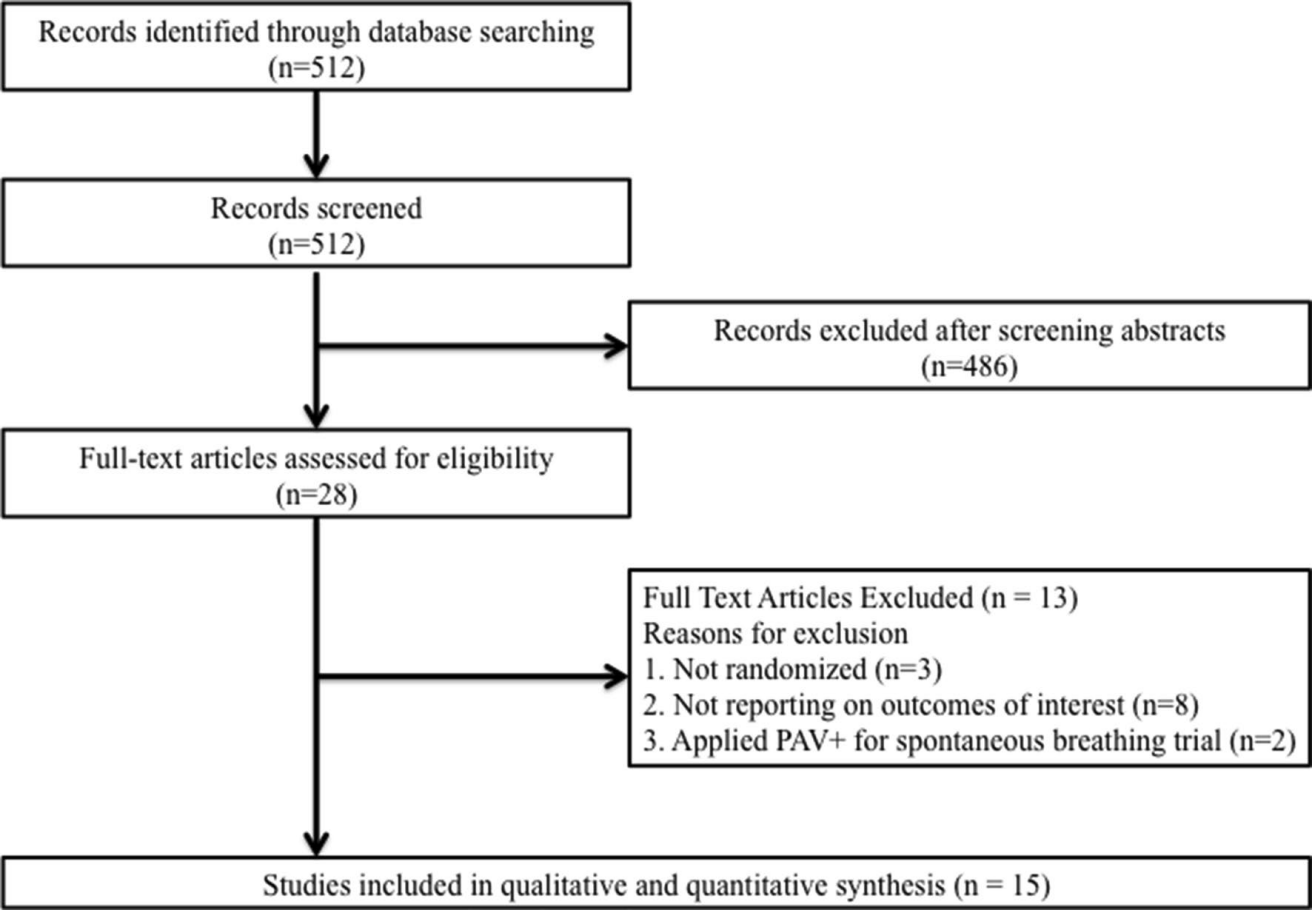
Study selection

The search extracted 512 articles. After application of our inclusion and exclusion criteria, we considered seven parallel-group [22–28] and eight crossover [15, 29–35] studies that compared proportional modes with PSV in mechanically ventilated adults (Fig. 1) (Additional file 2: Table S2). We contacted one study author to confirm that the participants were randomized, and therefore included this study in our analysis [33]. A total of 668 mechanical ventilated adult patients were included in the analysis (Table 1). The median or mean age of the participants ranged from 55.4 to 77.9 years, and 34.1% were women. Four studies evaluated PAV (*n *= 367) [22–24, 28], ten studies evaluated NAVA (*n *= 285) [25–27, 29–35], and one crossover study evaluated both modes (*n *= 16) [15]. We included the whole literature on proportional assist ventilation. However, we could not find studies that met our inclusion criteria other than examined PAV+. One parallel study recruited multicenter ICUs [25], six studies recruited medical ICUs [23, 27, 31–33, 35], one crossover study recruited surgical ICU [34], and seven studies recruited mixed (medical and surgical) ICUs [15, 22, 24, 26, 28–30]. All studies were published in full texts and in English.

**Table 1:** Characteristics of included studies

Study/year	Country	Study type	Type of ICU/inclusion criteria	Sample size (% female)	Mean or median age	Type of experiment	Intervention	Control
Colombo/2008 [29]	Italy	Crossover	Mixed ICU/patients on partial ventilator support	14 (14.2)	55.4	Physiological/20 min	NAVA (support level was to obtain similar peak inspiratory pressure to the PSV)	PSV (support level was to obtain VT of 6–8 ml/kg PBW)
Xirouchaki/2008 [22]	Greece	Parallel	Mixed ICU/patients on CMV > 36 h and awaiting weaning	208 (33.7)	59–63	Clinical/Weaning (48 h) (weaning failure was defined as the need for switching to a controlled mode or reintubation after extubation)	PAV+	PSV
Spahija/2010 [30]	Canada	Crossover	Mixed ICU/patients on MV for ARF and ready to be wean	14 (57.1)	69.4	Physiological/10 min	NAVA (support level was to obtain similar peak inspiratory pressure to the PSV)	PSV (support level was to obtain VT of 6–8 ml/kg PBW)
Patroniti/2012 [31]	Italy	Crossover	Medical ICU/patients on MV for ARF	15 (40)	67.9	Physiological/10 min	NAVA (support level was 1 cm H_2_O/μV)	PSV (support level was 8 cm H_2_O)
Elganady/2014 [23]	Egypt	Parallel	Medical ICU/patients with acute exacerbation of COPD and on MV > 24 h	60 (18.3)	59.7	Clinical/weaning (weaning failure was defined as the need for switching to a controlled mode or reintubation after extubation)	PAV+	PSV
Doorduin/2015 [32]	Netherlands	Crossover	Medical ICU/patients on MV for ARDS	12 (16.7)	64.1	Physiological/30 min	NAVA (support level was to obtain similar peak inspiratory pressure to the PSV)	PSV (support level was to obtain VT of 6 ml/kg PBW)
Schmidt/2015 [15]	France	Crossover	Mixed ICU/patients on MV > 48 h for ARF and awaiting weaning	16 (37.5)	67	Physiological/30 min	PAV+/NAVA (support level was to obtain VT of 6–8 ml/kg PBW)	PSV (support level was to obtain VT of 6–8 ml/kg PBW)
Bosma/2016 [24]	Canada	Parallel	Mixed ICU/MV > 36 h, SBT failure	50 (50)	64.8	Clinical/weaning (weaning failure was not defined)	PAV+	PSV
Carteaux/2016 [33]	France	Crossover	Medical ICU/patients on MV and recovering from ARF	11 (54.5)	69.8	Physiological/5–10 min	NAVA (support level was 0.5 cm H_2_O/μV)	PSV (support level was 10 cm H_2_O)
Demoule/2016 [25]	France	Parallel	Multicenter (medical/mixed/surgical)/patients who required endotracheal MV > 24 h for ARF and awaiting weaning	128 (32.8)	64–66	Clinical/Weaning (14 days) (weaning failure was defined as the need for switching to a controlled mode)	NAVA	PSV
Di Mussi/2016 [26]	Italy	Parallel	Mixed ICU/patients requiring CMV > 72 h for ARF	25 (44)	68.2	Physiological/48 h	NAVA (support level was titrated according to an initial steep increase P_AO_ and VT)	PSV (support level was to obtain VT of 5–8 ml/kg PBW)
Kuo/2016 [27]	Taiwan	Parallel	Medical ICU/patients aged ≥ 45 years, and received MV > 21 days for COPD exacerbation	33 (27.3)	77.9	Clinical/weaning (weaning failure was not defined)	NAVA	PSV
Costa/2017 [34]	Italy	Crossover	Surgical ICU/patients on partial ventilator support < 72 h	13 (23.1)	58.9	Physiological/25 min	NAVA (support level was to obtain similar VT and EAdi to the PSV)	PSV (support level was to obtain VT of 6–8 ml/kg PBW)
Ferreira/2017 [35]	Brazil	Crossover	Medical ICU/patients on MV > 48 h and considered to be ready for SBT	20 (35)	60	Physiological/30 min (SBT)	NAVA (support level was to obtain similar peak inspiratory pressure to the PSV)	PSV (support level was 5 cm H_2_O)
Botha/2018 [28]	Australia	Parallel	Mixed ICU/patients on CMV and suitable for > 48 h of weaning on a spontaneous mode	49 (40.8)	63	Clinical/weaning (weaning failure was defined as the need for reintubation after extubation)	PAV+	PSV

*ICU* intensive care unit, *PAV* proportional assist ventilation, *NAVA* neurally adjusted ventilatory assist, *PSV* pressure support ventilation, *MV* mechanical ventilation, *COPD* chronic obstructive pulmonary disease, *ARF* acute respiratory failure, *ARDS* acute respiratory distress syndrome, *SBT* spontaneous breathing trial, *NR* not reported, *CMV* controlled mechanical ventilation, *SBT* spontaneous breathing trial, *VT* tidal volume, *EAdi* diaphragm electrical activity

### Risk of bias

Sequence generation and allocation concealment were adequately conducted in ten (67%) [15, 22–25, 28, 29, 33–35] and five studies (33%) [23, 25, 28, 34, 35], respectively (Table 2). Blinding of participants and investigators was impossible due to the nature of study design, but blinding of outcome assessors was deemed appropriate in two studies (13%) [25, 35]. Nine studies (60%) were free of the risk of incomplete outcome reporting [22, 24, 27–29, 31, 33–35]. Since many of the included studies were crossover studies that failed to report that baseline data between groups, only four studies (27%) were considered at low risk of other bias [22–25].

**Table 2:** Risk of bias in included studies

Study/year	Sequence generation	Allocation concealment	Blinding of participants and personnel	Blinding of outcome assessors	Incomplete outcome data	Selective outcome reporting	Other source of bias
Colombo/2008	Low	Unclear	High	Unclear	Low	Unclear	Unclear
Xirouchaki/2008	Low	Unclear	High	Unclear	Low	Low	Low
Spahija/2010	Unclear	Unclear	High	Unclear	Unclear	Unclear	Unclear
Patroniti/2012	Unclear	Unclear	High	Unclear	Low	Unclear	Unclear
Elganady/2014	Low	Low	High	Unclear	Unclear	Low	Low
Doorduin/2015	Unclear	Unclear	High	Unclear	Unclear	Low	Unclear
Schmidt/2015	Low	Unclear	High	Unclear	Unclear	Low	Unclear
Bosma/2016	Low	Unclear	High	Unclear	Low	Unclear	Low
Carteaux/2016	Low	Unclear	High	High	Low	Low	Unclear
Demoule/2016	Low	Low	High	Low	High	Low	Low
Di mussi/2016	Unclear	Unclear	High	Unclear	High	High	Unclear
Kuo/2016	Unclear	Unclear	High	Unclear	Low	Low	Unclear
Costa/2017	Low	Low	High	Unclear	Low	Low	Unclear
Ferreira/2017	Low	Low	High	Low	Low	Low	Unclear
Botha/2018	Low	Low	High	High	Low	Low	High

### Primary outcomes

#### Asynchrony index

**Fig. 2:**
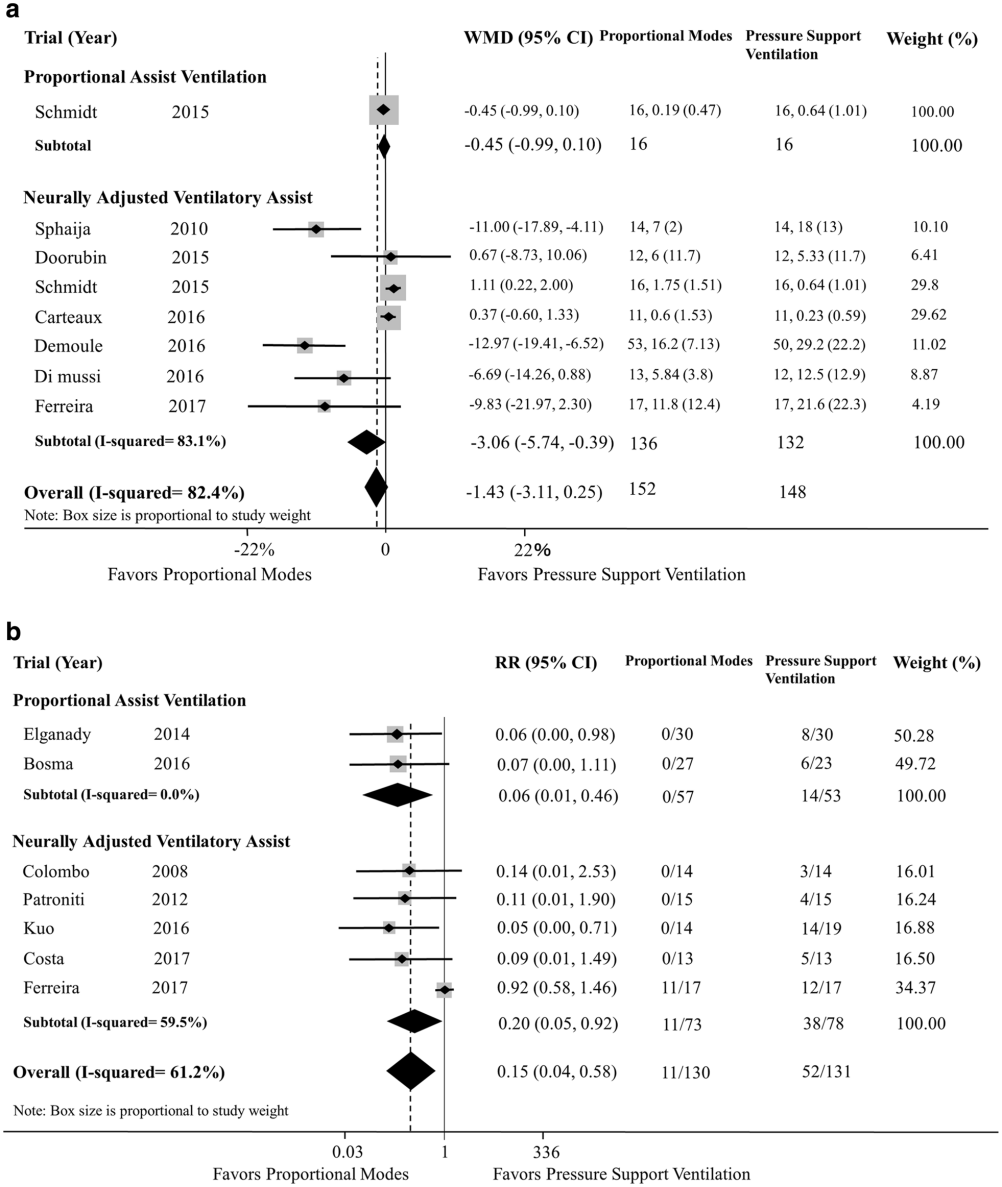
Relative risk of asynchrony index (AI) in included studies (**a** AI as a continuous outcome, **b** AI as dichotomous whenever the number of patients with AI > 10%). *RR* risk ratio

Although the use of proportional modes was not associated with a reduction in AI (WMD − 1.43; 95% CI − 3.11 to 0.25; *p *= 0.096; *df *= 7; *I*^2^ = 82.4%) (Fig. 2a), the use of proportional modes was associated with a reduction in patients with AI > 10% (RR 0.15; 95% CI 0.04–0.58; *p *= 0.006; *df *= 6; *I*^2^ = 61.2%) (Fig. 2b), compared with PSV. In the subgroup analysis, the use of NAVA was associated with a reduction in AI. There was a significant heterogeneity among studies for AI, especially with NAVA.

#### Weaning failure

**Fig. 3:**
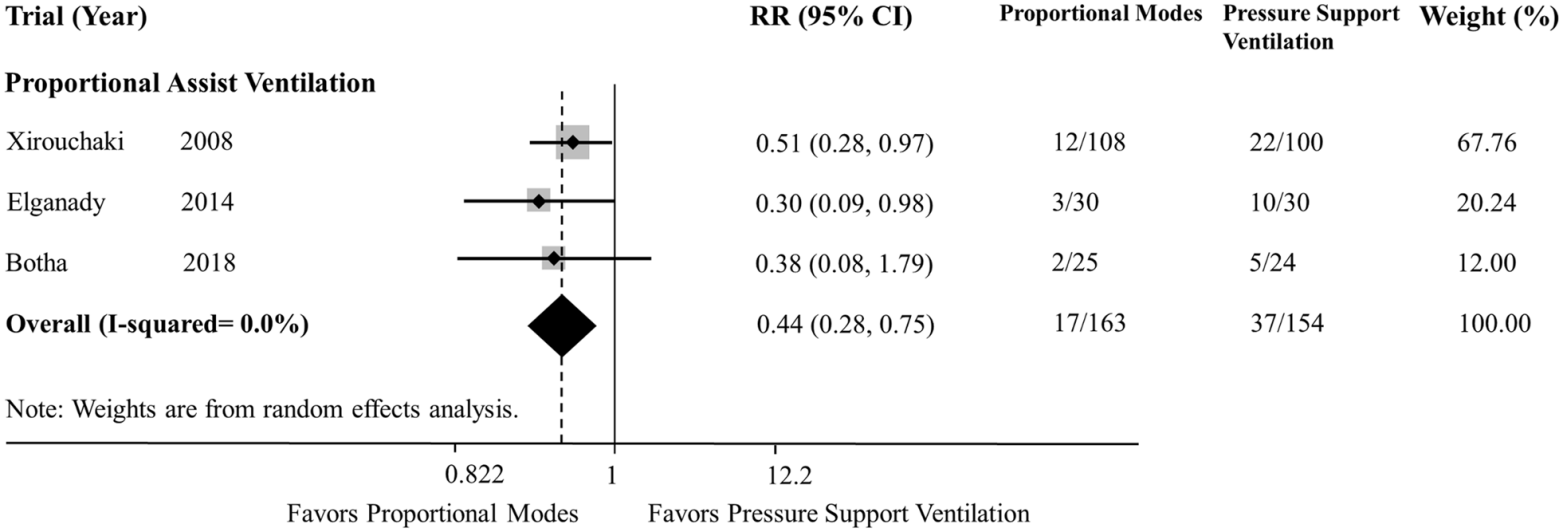
Relative risk of weaning failure in included studies. *RR* risk ratio

Three studies were included in the analysis, and all of them compared PAV and PSV [22, 23, 28]. The definition of weaning failure in each study is shown in Table 1. The use of proportional modes was associated with reduction in weaning failure (RR 0.44; 95% CI 0.26–0.75; *p *= 0.003; *df *= 2; *I*^2^ = 0.0%) (Fig. 3), compared with PSV.

#### Duration of mechanical ventilation

**Fig. 4:**
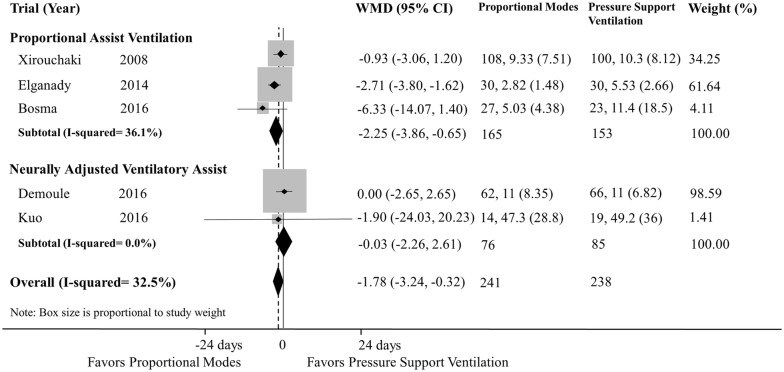
Relative risk of duration of mechanical ventilation in included studies. *RR* risk ratio

Five studies reported duration of mechanical ventilation (three and two studies evaluated PAV and NAVA, respectively) [22–25, 27]. The use of proportional modes was associated with a shorter duration of mechanical ventilation (WMD − 1.78 days; 95% CI − 3.24 to − 0.32; *p *= 0.017; *df *= 4; *I*^2^ = 32.5%) in comparison with PSV (Fig. 4). In the subgroup analysis, the use of PAV was associated with a reduction in duration of mechanical ventilation in comparison with PSV, while there was no such association for NAVA.

### Secondary outcomes

Compared with PSV, proportional modes did not exhibit any association with reduction in weaning time from randomization, switching again to a controlled mode, length of ICU stay, length of hospital stay, hospital mortality or tracheostomy, compared with use of PSV (Table 3). However, the use of proportional modes was significantly associated with reduced incidence of reintubation (RR 0.39; 95% CI 0.17–0.90; *p *= 0.027; *df *= 2; *I*^2^ = 0.0%) and the use of noninvasive ventilation after extubation (RR 0.64; 95% CI 0.47–0.89; *p *= 0.007; *df *= 1; *I*^2^ = 0.0%) in comparison with PSV (Table 3).

**Table 3:** Results of secondary outcomes

Outcomes	No. of trials (PAV/NAVA)	Total sample size	Summary estimates (95% confidence intervals)	*Q*	*df*	*I*^2^ (%)
Weaning time from randomization (day)	PAV 2 [24, 28] NAVA 1 [25]	220	WMD − 1.21 (− 4.32, 1.91)	3.66	2	45.3
Switching again to a controlled mode	PAV 1 [24] NAVA 1 [25]	168	RR 1.00 (0.77, 1.31)	0.30	1	0.0
Length of ICU (day)	PAV 4 [22–24, 28] NAVA 2 [25, 27]	528	WMD − 1.41 (− 3.90, 1.09)	12.43	5	59.8
Length of hospital stay (day)	PAV 3 [23, 24, 28] NAVA 2 [25, 27]	320	WMD − 0.26 (− 3.90, 3.37)	7.73	4	48.2
Tracheostomy	PAV 2 [24, 28]	98	RR 0.65 (0.31, 1.37)	0.09	1	0.0
Reintubation	PAV 3 [23, 24, 28]	158	RR 0.39 (0.17, 0.90)	0.39	2	0.0
Use of NIV after extubation	PAV 1 [24] NAVA 1 [25]	178	RR 0.64 (0.47, 0.89)	0.13	1	0.0

*RR* risk ratio, *WMD* weighted mean difference, *ICU* intensive care unit, *PAV* proportional assisted ventilation, *NAVA* neurally adjusted ventilatory assist

### Subgroup analysis

We conducted subgroup analyses on the primary outcomes (Table 4). There was no significant difference between NAVA and PAV groups in any of the primary outcomes.

**Table 4:** Subgroup analyses of primary outcomes

Outcomes	No. of trials (PAV/NAVA)	Total sample size	Summary estimates (95% confidence intervals)	Heterogeneity			*p* value (test for subgroup difference)
				*Q*	*df*	*I*^2^ (%)	
AI as continuous outcome	PAV, 1	32	WMD − 0.45 (− 1.00, 0.10)	0.00	0	–	0.06
	NAVA, 7	268	WMD − 3.06 (− 5.74, − 0.39)	35.53	6	83.1	
AI > 10%	PAV, 2	110	RR 0.06 (0.01, 0.46)	0.00	1	0.0	0.57
	NAVA, 5	151	RR 0.20 (0.05, 0.92)	15.47	4	59.5	
Weaning failure	PAV, 3	317	RR 0.44 (0.26, 0.75)	0.60	2	0.0	NA
Duration of mechanical ventilation	PAV, 3	318	WMD − 2.25 (− 3.86, − 0.65)	3.13	2	36.5	0.16
	NAVA, 2	161	WMD − 0.03 (− 2.66, 2.61)	0.03	1	0.0	

*AI* asynchrony index, *RR* risk ratio, *WMD* weighed mean difference

### Sensitivity analysis

We conducted sensitivity analyses on the primary outcomes (Additional file 3: Table S3). Although some sensitivity analysis was impossible for secondary outcomes due to the lack of low-risk studies, other available sensitivity analyses were mostly consistent with the primary outcome analysis. Pooling only one comparison from a three-way crossover study for AI yielded finding similar to the primary analysis.

## Discussion

The present study provides the following findings: (1) proportional modes reduced the incidence with AI > 10%, and (2) the use of proportional modes, especially PAV, was associated with a reduction in weaning failure and duration of mechanical ventilation, compared with PSV. Sensitivity analyses corroborated the robustness of our findings, and despite the small sample size within each of the included studies, our systematic review and meta-analysis suggests that proportional modes may have some merits for patients undergoing liberation from mechanical ventilation, compared with PSV.

Our study showed that proportional modes significantly reduced the incidence with AI > 10%, compared to PSV. The significant reduction of the incidence with AI > 10% is mainly attributed to the existence of neuromuscular coupling in proportional modes, allowing the patient to have control over the airway pressure provided by the ventilator according to the patient’s inspiratory demand. Over-assistance with PSV generated an important prevalence of ineffective effort. For instance, Schmidt et al. showed that PAV and NAVA improved patient–ventilator interaction by preventing over-distention and ineffective effort in their preliminary crossover study [15]. Although several patient–ventilator asynchronies classifications exist [36], we were unable to identify which classifications of asynchrony actually improved with each mode because AI was solely utilized as an outcome. PAV and NAVA may have different profiles in preventing asynchrony such as double triggering which was observed more frequently in NAVA than in both PAV and PSV [15]. On the other hand, inspiratory trigger delay was observed less frequently in NAVA than in both PAV and PSV [15].

Remarkably, our study showed that the use of proportional modes, especially PAV, was significantly associated with reduced weaning failure and duration of mechanical ventilation. The possible explanations for the finding are as follows. First, the patients with PSV may have been given more frequent or higher doses of analgesics or sedatives due to higher incidence of asynchrony. Previous studies showed that proportional modes could not only improve sleep quality [37] but also decrease the dose of sedative medication [24] because of better patient–ventilator interaction. Maintaining light levels of sedation is shown to be associated with shorter duration of mechanical ventilation [38, 39]. Second, asynchronies such as ineffective effort and double triggering may have unfavorable effects on patients’ respiratory systems, which lead to longer duration of mechanical ventilation on PSV. Third, proportional modes may reduce risk of over- and under-assistance. PAV+ monitors the work of breathing and inspiratory respiratory effort of the patients [40], while NAVA monitors the electrical activity of diaphragm and thus may minimize diaphragmatic atrophy due to inactivity. In fact, over-assistance is a common scenario with PSV and leads to diaphragm atrophy, explaining the increased duration of mechanical ventilation [41, 42]. However, these assumptions have been somewhat controversial. Two multicenter RCTs are presently under way (NCT02447692, NCT01730794), and their results will presumably become available in the near future. Our meta-analysis supports, while awaiting the results of the RCTs, that it is reasonable to use proportional modes in the liberation process for mechanically ventilated adults.

Finally, we determined that proportional modes were associated with less frequent application of post-extubation noninvasive ventilation and reintubation. Each study did not include predefined criteria for post-extubation noninvasive ventilation and reintubation. Due to a significant heterogeneity among studies and an insufficient number of studies, further investigation seems warranted to better understand the impact of proportional modes on secondary outcomes.

Our study has several strengths. First, to the best of our knowledge, this represents the first systematic review and meta-analysis that examines the efficacy of proportional modes as weaning modes. A comprehensive search was conducted, and fifteen studies were included. This study could reveal the efficacy of proportional modes as weaning modes, compared to PSV. Second, this systematic review examined a broad array of outcomes and hence could propose future pertinent proportional modes investigations to be conducted not only on weaning but also post-extubation outcomes, namely the utilization of noninvasive ventilation and reintubation. Third, subgroup analysis (PAV or NAVA) could be conducted due to the large number of studies.

Our study also has some limitations. First, both PAV and NAVA were concurrently analyzed since both have similar objectives. However, PAV and NAVA have some differences and thus are not completely the same. For instance, PAV delivers the support proportionally to lung mechanics, while NAVA cannot [43]. Therefore, there may be some differences in outcomes between PAV and NAVA in patients with abnormal respiratory system mechanics. Although a comparison of PAV and NAVA is thus clinically relevant, we did not conduct a network meta-analysis to compare PAV and NAVA for the following reasons: (1) the number of studies included in each analysis was limited; (2) a network meta-analysis is not recommended when there were presumably clinical heterogeneity across studies; and (3) the subgroup analysis already found no statistical difference between the two subgroups. Second, each included study in this systematic review utilized the differential weaning protocol which may possibly affect the duration of mechanical ventilation. Third, there was a significant heterogeneity among studies for AI, especially with NAVA, but an insufficient number of studies precluded the analysis to investigate the source of this heterogeneity. Fourth, the risk of bias in many of the included studies was deemed high. However, although it was impossible to perform some sensitivity analyses due to the lack of studies at low risk of certain bias, most available sensitivity analyses produced findings similar to the primary analysis, which further made the analysis more rigorous. Fifth, many of the included studies were crossover studies. They have a theoretical risk that the efficacy of the first intervention may be overestimated or underestimated in comparison with that of the second one [44], because the patients were supposed to gradually improve during the weaning period considered in the included studies.

## Conclusion

The use of proportional modes was associated with a reduction in the incidence with AI > 10%, weaning failure and duration of mechanical ventilation, compared with PSV. However, reduced weaning failure and duration of mechanical ventilation were found with only PAV. Due to a significant heterogeneity among studies and an insufficient number of studies, further investigation seems warranted to better understand the impact of proportional modes.

## Additional files

**Additional file 1: Table S1.** Search strategy.

**Additional file 2: Table S2.** Full text articles excluded.

**Additional file 3: Table S3.** Sensitivity analyses of primary outcomes.

## Abbreviations

PSV: pressure support ventilation
PAV: proportional assist ventilation
NAVA: neurally adjusted ventilatory assist
RCT: randomized controlled trial
AI: asynchrony index
EAdi: electrical activity of the diaphragm
RR: risk ratio
CI: confidence interval
WMD: weighted mean difference
