# Supplementary material for: Proportional modes versus pressure support ventilation: a systematic review and meta-analysis
Source: Ann Intensive Care. 2018 Dec 10;8:123. doi: 10.1186/s13613-018-0470-y (PMC6288104; doi:10.1186/s13613-018-0470-y)
Supplement: Supplementary file 1 — Additional file 1: Table S1. Search strategy. [file 13613_2018_470_MOESM1_ESM.docx]

Additional file 1: Table S1. Search Strategy.

(((((groups[tiab] OR trial[tiab]) OR randomly[tiab]) OR randomized[tiab]) OR controlled clinical trial[pt]) OR randomized controlled trial[pt]) AND ((psv[All Fields] OR (("pressure"[MeSH Terms] OR "pressure"[All Fields]) AND support[All Fields] AND ("ventilation"[MeSH Terms] OR "ventilation"[All Fields] OR "respiration"[MeSH Terms] OR "respiration"[All Fields]))) AND ((("nava"[All Fields] OR ("neurally adjusted ventilatory assist"[All Fields] OR "neurally adjusted ventilator assist"[All Fields])) OR "pav"[All Fields]) OR ("interactive ventilatory support"[MeSH Terms] OR ("interactive"[All Fields] AND "ventilatory"[All Fields] AND "support"[All Fields]) OR "interactive ventilatory support"[All Fields] OR ("proportional"[All Fields] AND "assist"[All Fields] AND "ventilation"[All Fields]) OR "proportional assist ventilation"[All Fields])))
