# Supplementary material for: Proportional modes versus pressure support ventilation: a systematic review and meta-analysis
Source: Ann Intensive Care. 2018 Dec 10;8:123. doi: 10.1186/s13613-018-0470-y (PMC6288104; doi:10.1186/s13613-018-0470-y)
Supplement: Supplementary file 2 — Additional file 2: Table S2. Full text articles excluded. [file 13613_2018_470_MOESM2_ESM.docx]

Additional file 2: Table S2. Full Text Articles Excluded.

1. **Not randomized PAV/NAVA or PSV (n=3)**

- [Piquilloud L](https://www.ncbi.nlm.nih.gov/pubmed/?term=Piquilloud%20L%5BAuthor%5D&cauthor=true&cauthor_uid=20871978), [Vignaux L](https://www.ncbi.nlm.nih.gov/pubmed/?term=Vignaux%20L%5BAuthor%5D&cauthor=true&cauthor_uid=20871978), [Bialais E](https://www.ncbi.nlm.nih.gov/pubmed/?term=Bialais%20E%5BAuthor%5D&cauthor=true&cauthor_uid=20871978), et al. Neurally adjusted ventilatory assist improves patient-ventilator interaction. Intensive Care Med. 2011;37:263-71.
- [Costa R](https://www.ncbi.nlm.nih.gov/pubmed/?term=Costa%20R%5BAuthor%5D&cauthor=true&cauthor_uid=21720909), [Spinazzola G](https://www.ncbi.nlm.nih.gov/pubmed/?term=Spinazzola%20G%5BAuthor%5D&cauthor=true&cauthor_uid=21720909), [Cipriani F](https://www.ncbi.nlm.nih.gov/pubmed/?term=Cipriani%20F%5BAuthor%5D&cauthor=true&cauthor_uid=21720909), et al. A physiologic comparison of proportional assist ventilation with load-adjustable gain factors (PAV+) versus pressure support ventilation (PSV). Intensive Care Med. 2011;37:1494-500.
- [Vasconcelos RS](https://www.ncbi.nlm.nih.gov/pubmed/?term=Vasconcelos%20RS%5BAuthor%5D&cauthor=true&cauthor_uid=28196936), [Sales RP](https://www.ncbi.nlm.nih.gov/pubmed/?term=Sales%20RP%5BAuthor%5D&cauthor=true&cauthor_uid=28196936), [Melo LHP](https://www.ncbi.nlm.nih.gov/pubmed/?term=Melo%20LHP%5BAuthor%5D&cauthor=true&cauthor_uid=28196936), et al. Influences of Duration of Inspiratory Effort, Respiratory Mechanics, and Ventilator Type on Asynchrony With Pressure Support and Proportional Assist Ventilation. Respir Care. 2017;62:550-557.

1. **Not reporting on outcomes of interests (n=8)**

- [Giannouli E](https://www.ncbi.nlm.nih.gov/pubmed/?term=Giannouli%20E%5BAuthor%5D&cauthor=true&cauthor_uid=10351909), [Webster K](https://www.ncbi.nlm.nih.gov/pubmed/?term=Webster%20K%5BAuthor%5D&cauthor=true&cauthor_uid=10351909), [Roberts D](https://www.ncbi.nlm.nih.gov/pubmed/?term=Roberts%20D%5BAuthor%5D&cauthor=true&cauthor_uid=10351909), et al. Response of ventilator-dependent patients to different levels of pressure support and proportional assist. Am J Respir Crit Care Med. 1999;159:1716-25.
- [Bosma K](https://www.ncbi.nlm.nih.gov/pubmed/?term=Bosma%20K%5BAuthor%5D&cauthor=true&cauthor_uid=17334259), [Ferreyra G](https://www.ncbi.nlm.nih.gov/pubmed/?term=Ferreyra%20G%5BAuthor%5D&cauthor=true&cauthor_uid=17334259), [Ambrogio C](https://www.ncbi.nlm.nih.gov/pubmed/?term=Ambrogio%20C%5BAuthor%5D&cauthor=true&cauthor_uid=17334259), et al. Patient-ventilator interaction and sleep in mechanically ventilated patients: pressure support versus proportional assist ventilation. Crit Care Med. 2007;35:1048-54.
- [Coisel Y](https://www.ncbi.nlm.nih.gov/pubmed/?term=Coisel%20Y%5BAuthor%5D&cauthor=true&cauthor_uid=20823760), [Chanques G](https://www.ncbi.nlm.nih.gov/pubmed/?term=Chanques%20G%5BAuthor%5D&cauthor=true&cauthor_uid=20823760), [Jung B](https://www.ncbi.nlm.nih.gov/pubmed/?term=Jung%20B%5BAuthor%5D&cauthor=true&cauthor_uid=20823760), et al. Neurally adjusted ventilatory assist in critically ill postoperative patients: a crossover randomized study. Anesthesiology. 2010;113:925-35.
- [Alexopoulou C](https://www.ncbi.nlm.nih.gov/pubmed/?term=Alexopoulou%20C%5BAuthor%5D&cauthor=true&cauthor_uid=23417203), [Kondili E](https://www.ncbi.nlm.nih.gov/pubmed/?term=Kondili%20E%5BAuthor%5D&cauthor=true&cauthor_uid=23417203), [Plataki M](https://www.ncbi.nlm.nih.gov/pubmed/?term=Plataki%20M%5BAuthor%5D&cauthor=true&cauthor_uid=23417203), [et al](https://www.ncbi.nlm.nih.gov/pubmed/?term=Georgopoulos%20D%5BAuthor%5D&cauthor=true&cauthor_uid=23417203). Patient-ventilator synchrony and sleep quality with proportional assist and pressure support ventilation. Intensive Care Med. 2013;39:1040-7.
- [Vagheggini G](https://www.ncbi.nlm.nih.gov/pubmed/?term=Vagheggini%20G%5BAuthor%5D&cauthor=true&cauthor_uid=24035203), [Mazzoleni S](https://www.ncbi.nlm.nih.gov/pubmed/?term=Mazzoleni%20S%5BAuthor%5D&cauthor=true&cauthor_uid=24035203), [Vlad Panait E](https://www.ncbi.nlm.nih.gov/pubmed/?term=Vlad%20Panait%20E%5BAuthor%5D&cauthor=true&cauthor_uid=24035203), et al. Physiologic response to various levels of pressure support and NAVA in prolonged weaning. Respir Med. 2013;107:1748-54.
- [Vaschetto R](https://www.ncbi.nlm.nih.gov/pubmed/?term=Vaschetto%20R%5BAuthor%5D&cauthor=true&cauthor_uid=23982026), [Cammarota G](https://www.ncbi.nlm.nih.gov/pubmed/?term=Cammarota%20G%5BAuthor%5D&cauthor=true&cauthor_uid=23982026), [Colombo D](https://www.ncbi.nlm.nih.gov/pubmed/?term=Colombo%20D%5BAuthor%5D&cauthor=true&cauthor_uid=23982026), et al. Effects of propofol on patient-ventilator synchrony and interaction during pressure support ventilation and neurally adjusted ventilatory assist. Crit Care Med. 2014;42:74-82.
- [Gautam PL](https://www.ncbi.nlm.nih.gov/pubmed/?term=Gautam%20PL%5BAuthor%5D&cauthor=true&cauthor_uid=28149025), [Kaur G](https://www.ncbi.nlm.nih.gov/pubmed/?term=Kaur%20G%5BAuthor%5D&cauthor=true&cauthor_uid=28149025), [Katyal S](https://www.ncbi.nlm.nih.gov/pubmed/?term=Katyal%20S%5BAuthor%5D&cauthor=true&cauthor_uid=28149025), et al. Comparison of patient-ventilator asynchrony during pressure support ventilation and proportional assist ventilation modes in surgical Intensive Care Unit: A randomized crossover study. Indian J Crit Care Med. 2016;20:689-694.
- Akoumianaki E, [Dousse N](https://www.ncbi.nlm.nih.gov/pubmed/?term=Dousse%20N%5BAuthor%5D&cauthor=true&cauthor_uid=28608135), [Lyazidi A](https://www.ncbi.nlm.nih.gov/pubmed/?term=Lyazidi%20A%5BAuthor%5D&cauthor=true&cauthor_uid=28608135), et al. Can proportional ventilation modes facilitate exercise in critically ill patients? A physiological cross-over study : Pressure support versus proportional ventilation during lower limb exercise in ventilated critically ill patients. Ann Intensive Care. 2017;7:64.

1. **Applied PAV+ only for spontaneous breathing trial (n=2)**

- Sasikumar S, Shanbhag V, Shenoy A. Comparison of pressure support ventilation and proportional assist ventilation plus for weaning from mechanical ventilation in critically ill patients. Ind J Resp Care 2013; 2:292-8.
- Teixeira SN, Osaku EF, Costa CR, et al. Comparison of Proportional Assist Ventilation Plus, T-Tube Ventilation, and Pressure Support Ventilation as Spontaneous Breathing Trials for Extubation: A Randomized Study. Respir Care. 2015;60:1527-35.
