# Supplementary material for: Proportional modes versus pressure support ventilation: a systematic review and meta-analysis
Source: Ann Intensive Care. 2018 Dec 10;8:123. doi: 10.1186/s13613-018-0470-y (PMC6288104; doi:10.1186/s13613-018-0470-y)
Supplement: Supplementary file 3 — Additional file 3: Table S3. Sensitivity analyses of primary outcomes. [file 13613_2018_470_MOESM3_ESM.docx]

Additional file 3: Table S3. Sensitivity Analyses of Primary Outcomes

| Outcomes | No. of Trials | Total Sample Size | Summary Estimates  (95% Confidence Intervals) |  | | |
| --- | --- | --- | --- | --- | --- | --- |
|  |  |  |  | Q | df | I^2^, % |
| Analyses excluding trials with unclear or high risk of bias in the domain of sequence generation | | | | | | |
| AI as continuous outcome | 5 | 223 | WMD, -0.49 (-2.05, 1.06) | 27.15 | 4 | 85.3 |
| AI >10% | 5 | 198 | RR, 0.19 (0.04, 1.46) | 10.15 | 4 | 60.6 |
| Weaning Failure | 3 | 317 | RR, 0.44 (0.26, 0.75) | 0.60 | 2 | 0.0 |
| Duration of Mechanical Ventilation | 4 | 446 | WMD, -1.74 (-3.39, -0.08) | 5.93 | 3 | 49.4 |
| Analyses excluding trials with unclear or high risk of bias in the domain of allocation concealment | | | | | | |
| AI as continuous outcome | 2 | 139 | WMD, -12.28 (-17.97, -6.58) | 0.20 | 1 | 0.0 |
| AI >10% | 1 | 60 | RR, 0.06 (0.004, 0.98) | 0.00 | 0 | - |
| Weaning Failure | 2 | 109 | RR, 0.33 (0.13, 0.84) | 0.06 | 1 | 0.0 |
| Duration of Mechanical Ventilation | 2 | 188 | WMD, -1.64 (-4.24, 0.96) | 3.44 | 1 | 71.0 |
| Analyses excluding trials with unclear or high risk of bias in the domain of blinding of outcome assessors | | | | | | |
| AI as continuous outcome | 2 | 137 | WMD, -12.28 (-17.97, -6.58) | 0.20 | 1 | 0.0 |
| AI >10% | 1 | 34 | RR, 0.92 (0.58, 1.46) | 0.00 | 0 | - |
| Weaning Failure | 0 | - | - | - | - | - |
| Duration of Mechanical Ventilation | 1 | 128 | WMD, 0.00 (-2.65, 2.65) | 0.00 | 0 | - |
| Analyses excluding trials with unclear or high risk of bias in the domain of incomplete outcome reporting | | | | | | |
| AI as continuous outcome | 2 | 56 | WMD, -2.87 (-12.17, 6.44) | 2.70 | 1 | 62.9 |
| AI >10% | 6 | 201 | RR, 0.17 (0.04, 0.71) | 12.60 | 5 | 60. |
| Weaning Failure | 3 | 317 | RR, 0.44 (0.26, 0.75) | 0.60 | 2 | 0.0 |
| Duration of Mechanical Ventilation | 3 | 291 | WMD, -1.32 (-3.36, 0.73) | 1.74 | 2 | 0.0 |
| Analyses excluding trials with unclear or high risk of bias in the domain of selective outcome reporting | | | | | | |
| AI as continuous outcome | 5 | 225 | WMD, -1.25 (-3.15, 0.99) | 26.43 | 4 | 84.9 |
| AI >10% | 4 | 153 | RR, 0.17 (0.03, 1.14) | 10.04 | 3 | 70.1 |
| Weaning Failure | 2 | 257 | RR 0.48 (0.27, 0.88) | 0.10 | 1 | 0.0 |
| Duration of Mechanical Ventilation | 4 | 429 | WMD, -1.63 (-3.10, -0.15) | 4.77 | 3 | 37.2 |

Abbreviations; AI, asynchrony index; RR, risk ratio; WMD, weighed mean difference.
